# Fusion of augmented reality imaging with the endoscopic view for endonasal skull base surgery; a novel application for surgical navigation based on intraoperative cone beam computed tomography and optical tracking

**DOI:** 10.1371/journal.pone.0227312

**Published:** 2020-01-16

**Authors:** Marco Lai, Simon Skyrman, Caifeng Shan, Drazenko Babic, Robert Homan, Erik Edström, Oscar Persson, Gustav Burström, Adrian Elmi-Terander, Benno H. W. Hendriks, Peter H. N. de With

**Affiliations:** 1 Philips Research, Eindhoven, The Netherlands; 2 Eindhoven University of Technology (TU/e), Eindhoven, The Netherlands; 3 Department of Neurosurgery, Karolinska University Hospital and Department of Clinical Neuroscience, Karolinska Institutet, Stockholm, Sweden; 4 Philips Healthcare, Best, The Netherlands; 5 Department of Biomechanical Engineering, Delft University of Technology, Delft, The Netherlands; University of California San Francisco, UNITED STATES

## Abstract

**Objective:**

Surgical navigation is a well-established tool in endoscopic skull base surgery. However, navigational and endoscopic views are usually displayed on separate monitors, forcing the surgeon to focus on one or the other. Aiming to provide real-time integration of endoscopic and diagnostic imaging information, we present a new navigation technique based on augmented reality with fusion of intraoperative cone beam computed tomography (CBCT) on the endoscopic view. The aim of this study was to evaluate the accuracy of the method.

**Material and methods:**

An augmented reality surgical navigation system (ARSN) with 3D CBCT capability was used. The navigation system incorporates an optical tracking system (OTS) with four video cameras embedded in the flat detector of the motorized C-arm. Intra-operative CBCT images were fused with the view of the surgical field obtained by the endoscope’s camera. Accuracy of CBCT image co-registration was tested using a custom-made grid with incorporated 3D spheres.

**Results:**

Co-registration of the CBCT image on the endoscopic view was performed. Accuracy of the overlay, measured as mean target registration error (TRE), was 0.55 mm with a standard deviation of 0.24 mm and with a median value of 0.51mm and interquartile range of 0.39**˗˗**0.68 mm.

**Conclusion:**

We present a novel augmented reality surgical navigation system, with fusion of intraoperative CBCT on the endoscopic view. The system shows sub-millimeter accuracy.

## Introduction

Endoscopic endonasal skull base surgery offers a minimally-invasive approach to skull base pathologies, including tumors, infectious diseases, CSF leak, vascular and compressive conditions affecting the cranial fossae and sinuses. This technique has several potential advantages, such as shortened hospitalization, reduced postoperative pain and lower complication rates compared to open surgery [1, 2]. However, approaching the skull base from the nasal cavity implies that the surgical target and adjacent risk organs, such as the carotid arteries and cranial nerves, are covered by bone and not in direct view. Successful use of endoscopy requires surgical experience and in-depth knowledge of anatomical landmarks. To further increase safety, surgical navigation has been implemented and is today a well-established tool in endoscopic skull base surgery [3–6]. While some studies have failed to demonstrate a positive impact of navigation in endonasal surgery [7–9], others have shown reduced complication rates and improved patient outcome [10–15].

Available navigation systems in clinical use are based on co-registration of preoperative CT and MR images to a coordinate system with a fixed relation to the patient’s head. This allows tracking and visualization of a pointer tool, or other instrument, in relation to the patient and the preoperative imaging. The navigational feedback, showing the instrument in relation to the patient’s imaging anatomy, is displayed on a dedicated screen [16]. Thus, since endoscopy works through line of sight, there is consequently no real-time information on sub-surface structures. The use of a pointer tool also means that the surgery must be paused during navigation. It has been shown that navigation tends to increase OR time in endoscopic endonasal procedures [10, 15, 17–19].

In the past few decades augmented reality (AR) has been investigated as a method to improve endoscopic navigation. AR is a technique where real-world objects are enhanced by overlay of computer-generated perceptual information. In the case of endoscopic surgery, AR can be used to augment the live video stream from the endoscope with overlaid image data from pre- or intraoperative radiological exams, like MRI- or CT-scans. Thus, a computer-generated image of a pre-planned surgical target, path or risk organ, can be integrated in the endoscope’s real-world view. In this way, sub-surface structures can be visualized and a pointer tool is not needed [20–22]. AR navigation systems have been successfully applied in several surgical fields, including microsurgery and spine surgery [23–26].

The AR systems proposed for endoscopic surgery have thus far mostly relied on preoperative imaging and contour-based registration protocols, which may result in surgically insufficient accuracy [27]. A commercially available system with direct navigational feedback in the endoscopic view allowing the overlay of annotations and models, is the Target Guided Surgery System. This system supports both electromagnetic and optical tracking as well as simultaneous hybrid tracking and, as for other AR navigation systems, a contour-based protocol is used for preoperative CTs registration on endoscopic images. Surgical targets and pathways are depicted as geometric figures overlaid on the endoscopic view [28]. Since this protocol is quite different from the followed approach in our system, a detailed comparison is not relevant. Alternatively, navigation systems based on intraoperative cone beam computed tomography (CBCT) have proved to reach sub-millimeter accuracy in skull-base surgery [29]. In addition, intraoperative CBCT allows for acquisition of updated images during surgery [26, 30–33].

In this study, we present a novel navigation technique for endoscopic endonasal skull base surgery, based on an augmented reality surgical navigation (ARSN) system, previously presented in [20]. Integrating an endoscope into the system allows us to augment intraoperative CBCT imaging data onto the endoscope view during surgery. The aim of the study was to test the accuracy of the system.

## Material and methods

### The endoscope

A rigid endoscope was used (model 28132AA, straightforward telescope 0º, Karl Storz GmbH & Co. KG, Tuttlingen, Germany), and attached to a 5-Mpixel camera (model acA2500-14uc, Basler Beteiligungs-GmbH & Co. KG, Ahrensburg, Germany) via a 35-mm focal-length endoscope-camera coupler. Images of the endoscope camera were acquired at 15 fps (frames per second) and at a resolution of 2590x1942 pixels.

### Skull phantom

A skull phantom was used for simulating the workflow in a surgical scenario. The skull phantom model was downloaded from the Internet and 3D printed in-house in PLA plastic material. Also, two inserts that mimic the internal carotids (cylinders with diameter of 3 mm), one insert that mimics the optic nerve (cylinders with diameter of 2 mm) and one insert that mimics the pituitary gland (sphere with diameter of 10 mm) were 3D printed in a resin material and glued inside the skull. Afterwards, the head was fixed on a stable plastic base.

### The augmented reality surgical navigation system

**Fig 1:**
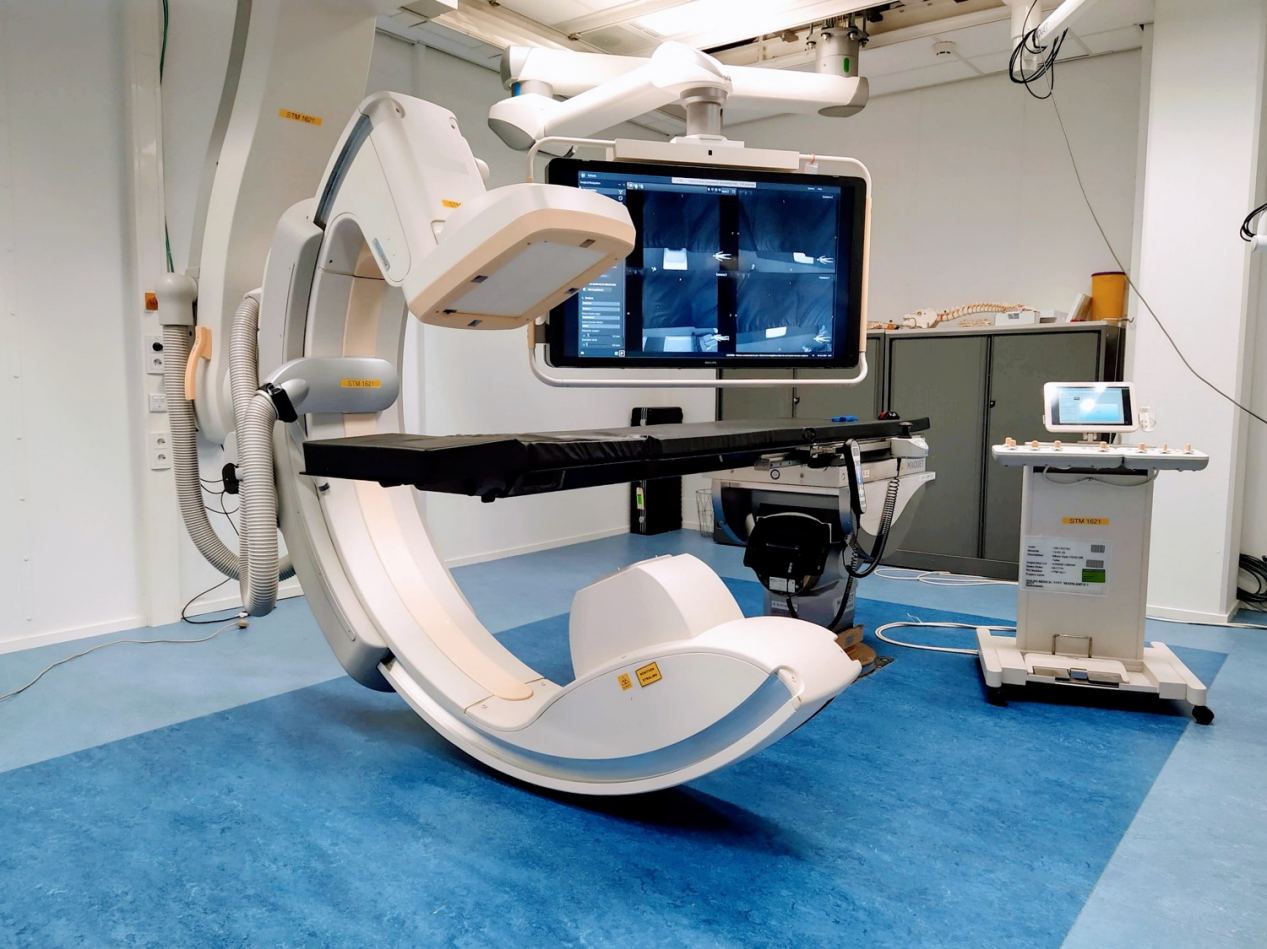
Augmented reality surgical navigation system for endoscopy.

**Fig 2:**
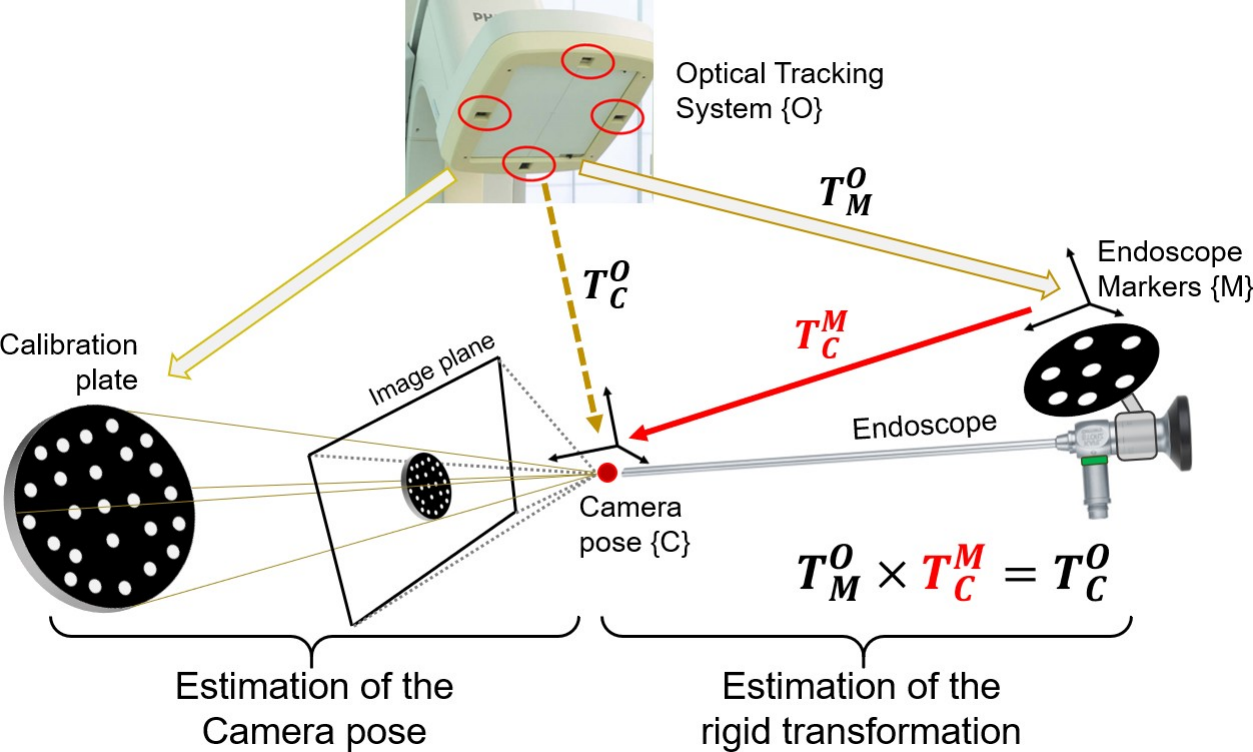
Experimental setup for the study on the skull phantom.

We present a new method for endoscope tracking and image augmentation, based on a previously presented augmented reality surgical navigation system (Philips Healthcare, Best, The Netherlands; Fig 1) [34]. The ARSN system has its own proprietary software for planning, segmentation and image processing. The system is composed of two parts: a C-arm for CBCT image acquisition and an optical tracking system (OTS), which makes use of four small high-resolution cameras in the flat-panel X-ray detector of the C-arm [20]. The use of four cameras increases robustness, since only two cameras are needed for marker detection and tracking. The OTS runs at 15 fps and tracks optical markers, each consisting of a 7-mm diameter white disk on a black background. The optical markers are automatically identified in the same coordinate system as the CBCT images. To allow this, a simple calibration procedure is performed when the system is set up by using several markers, which for this initial procedure are both optical and radiopaque and therefore are seen by the OTS and recognized on the CBCT images. This calibration creates a rigid integration of the two parts of the ARSN system and does not need to be repeated. For endoscope tracking and image augmentation (Fig 2), however, the following steps are performed for every surgical procedure.

1. Endoscope calibration
2. CBCT acquisition and co-registration with OTS
3. *Image fusion on the endoscopic view*.

### 1. Endoscope calibration

**Fig 3:**
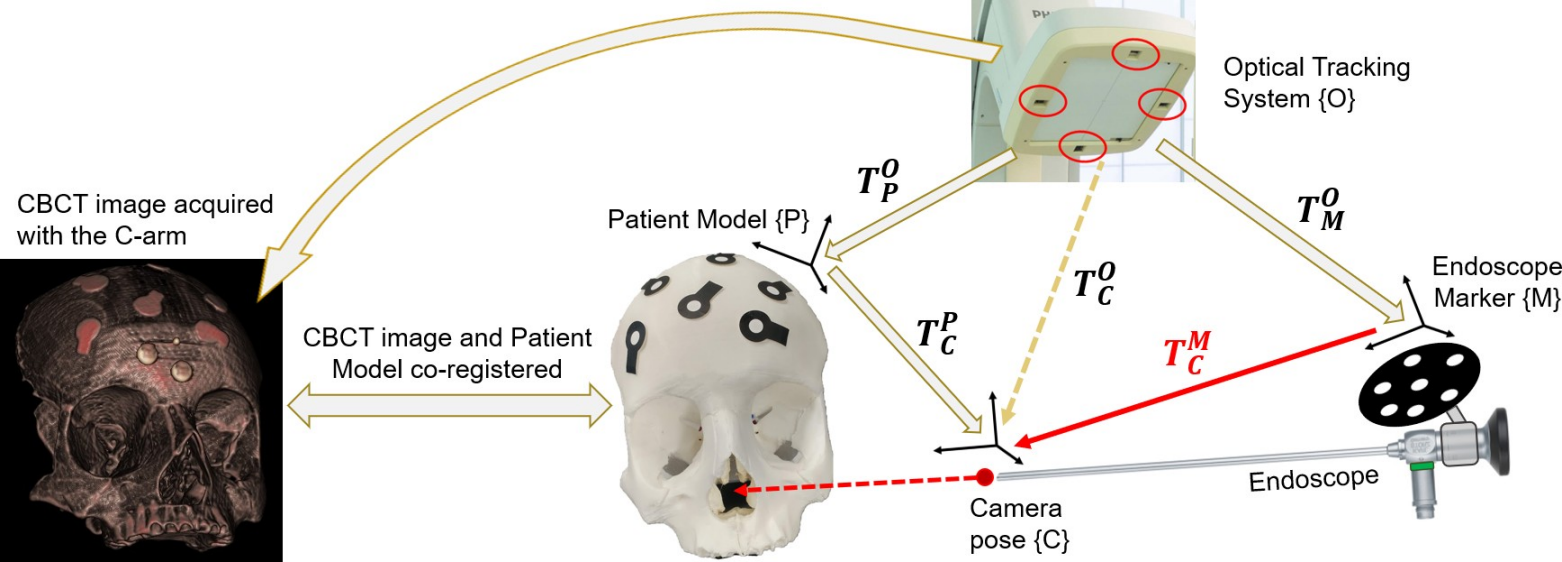
Hand-eye calibration with a moving calibration plate.

First, an endoscope marker (EM), a 5-cm diameter aluminium disc with a printed pattern of optical markers, was attached to the collar of the endoscope for detection and tracking by the OTS. Second, the intrinsic endoscope camera parameters were computed with the Zhang algorithm [35], using 15 images of a checkerboard at multiple perspectives. Third, extrinsic parameters were computed using a hand-eye camera calibration algorithm [36], which defined the rigid transformation $T_{C}^{M}$ between the EM mounted on the endoscope $T_{M}^{O}$, tracked via the OTS, and the camera pose $T_{C}^{O}$ (Fig 3). For this, a calibration-plate (CP), with a pattern of 25 optical markers for the OTS was used. The endoscope was fixed in position by a surgical arm, while the CP was moved manually. Twenty views of the EM and the CP were acquired with the OTS while the CP was photographed with the endoscope. For each of the twenty views, the camera pose $T_{C}^{O}$ were calculated with the P3P (Perspective 3 Points) algorithm [37], combining the 3D marker locations of the CP and their corresponding 2D endoscopic image projections, as well as the calibrated intrinsic endoscope-camera parameters. A dataset of EM-positions as detected by the OTS and the relative camera poses was constructed and, eventually, the rigid transformation $T_{C}^{M}$ was then computed, following a least-square minimization method [36].

### 2. CBCT acquisition and co-registration with OTS

A skull phantom was positioned on the surgical table and 5–10 optical markers were placed on its surface and tracked by the cameras. The detected optical markers generated a virtual reference grid (VRG) on the skull surface that was constantly tracked by the OTS. A CBCT image of the skull phantom was acquired, during which the VRG was co-registered with the CBCT image. At this point, any movement recognized by the OTS could be compensated for in the CBCT 3D volume.

### 3. Image fusion on the endoscopic view

**Fig 4:**
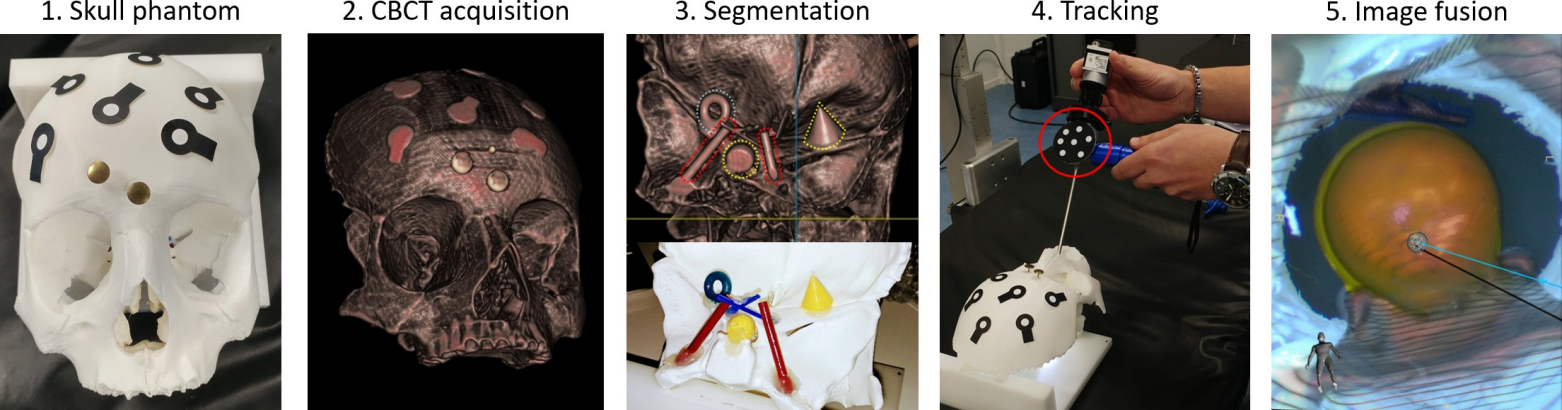
Relationship of the frame transformations.

The CBCT image could be overlaid on the endoscopic image, as shown in Fig 4, by defining the transformation $T_{C}^{P}$ from the patient model $T_{P}^{O}$ to the camera position and orientation (i.e. pose) $T_{C}^{O}$, such that:

$$T_{C}^{P}={T^{-1}}_{P}^{O}\times T_{C}^{O}.$$

The patient model $T_{P}^{O}$ was defined based on the optical markers placed on the surface of the skull phantom and their resulting, VRG. Using the VRG, the CBCT image could be adjusted according to the motion of the skull phantom $T_{P}^{O}$. The camera pose $T_{C}^{O}$ was defined as:

$$T_{C}^{O}=T_{M}^{O}\times T_{C}^{M},$$

with $T_{M}^{O}$ pose of the MP tracked via the OTS and $T_{C}^{M}$ the rigid transformation computed during the hand-eye calibration step. The complete transformation which expresses the skull phantom in the camera position reference system can be written as:

$$T_{C}^{P}={T^{-1}}_{P}^{O}\times (T_{M}^{O}{\times T}_{C}^{M}).$$

**Fig 5:**
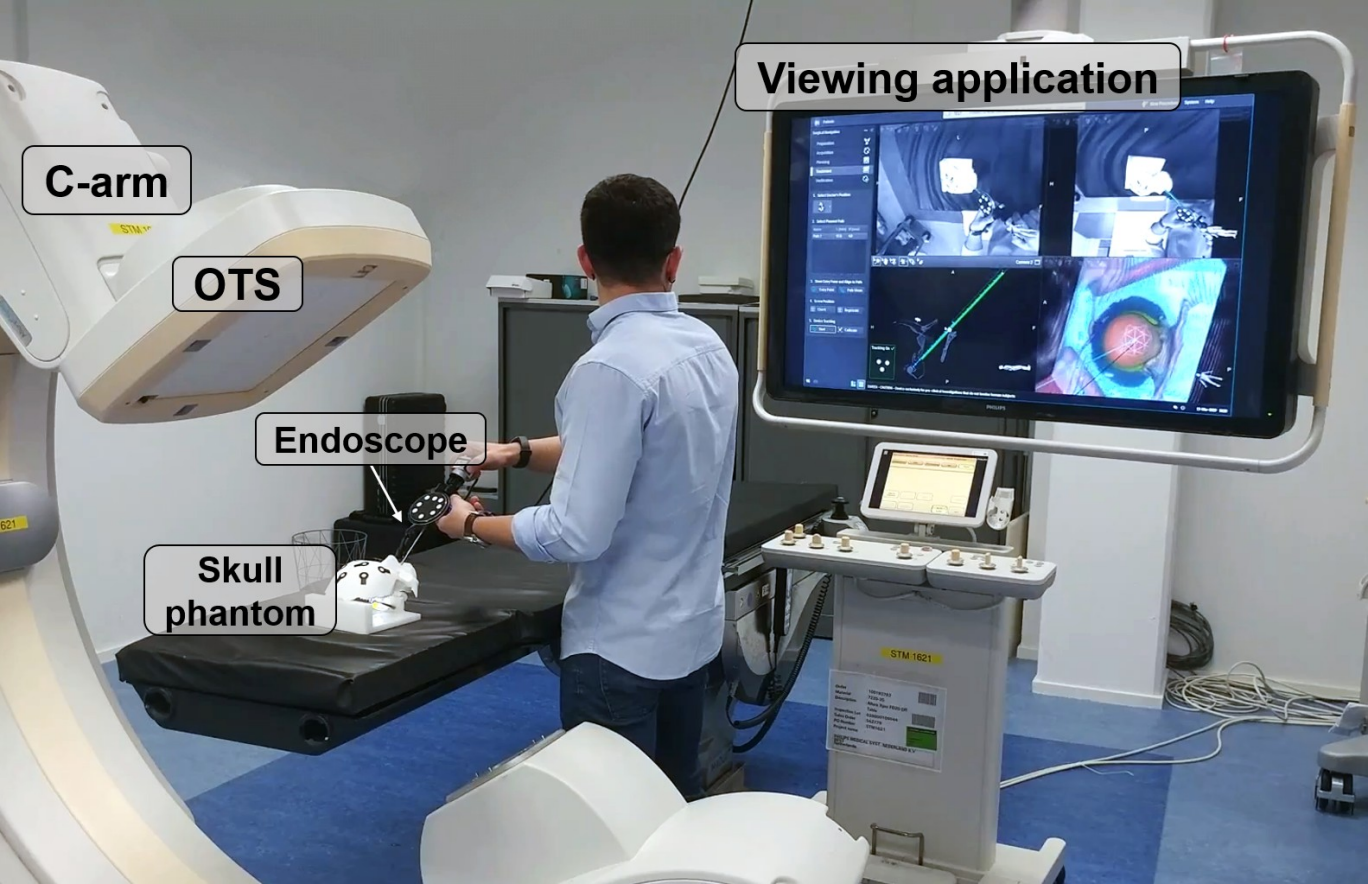
The workflow in a surgical scenario. Overall performances of the image fusion system were evaluated on a plastic skull phantom with a realistic representation of the nasal cavity and adjacent skull base anatomy, including vessels, nerves and the pituitary gland. 1. The skull phantom with optical markers on its surface was positioned on the surgical table. The 3D position of the optical markers was detected by the OTS of the navigation system, to create a VRG for tracking of the phantom’s motion. 2. A CBCT image, co-registered with the 3D position of the optical markers (VRG) was acquired. 3. Anatomical structures of interest were manually segmented from the CBCT image. 4. The endoscope, automatically recognized and tracked by the OTS, was placed in the nasal cavity of the phantom. 5. Segmented structures at the base of the skull were augmented onto the live endoscopic image. The augmented endoscopic view, together with anatomical views to guide the surgeon inside the nasal cavity, were displayed.

**Fig 6:**
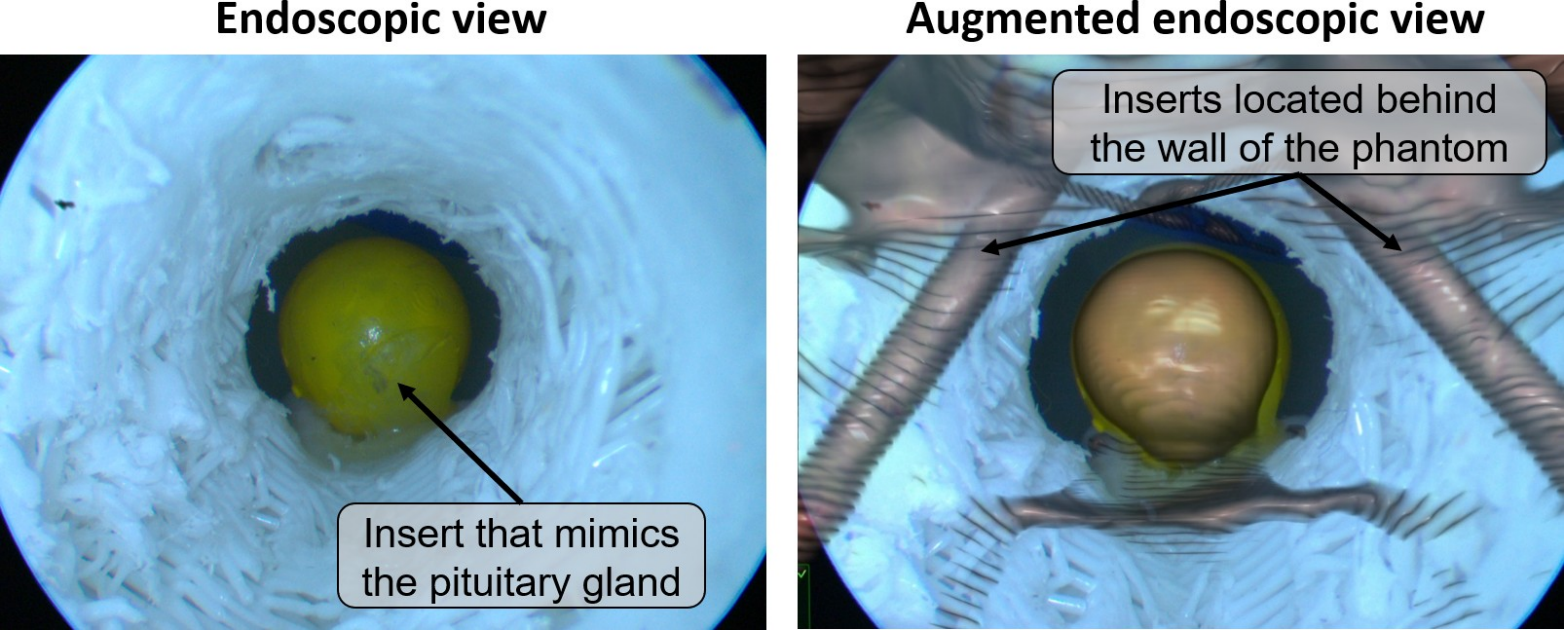
Example of image fusion on the endoscopic view.

This series of transformations lead to the co-registration of CBCT and the endoscopic image (Figs 5 and 6).

### Test of accuracy

**Fig 7:**
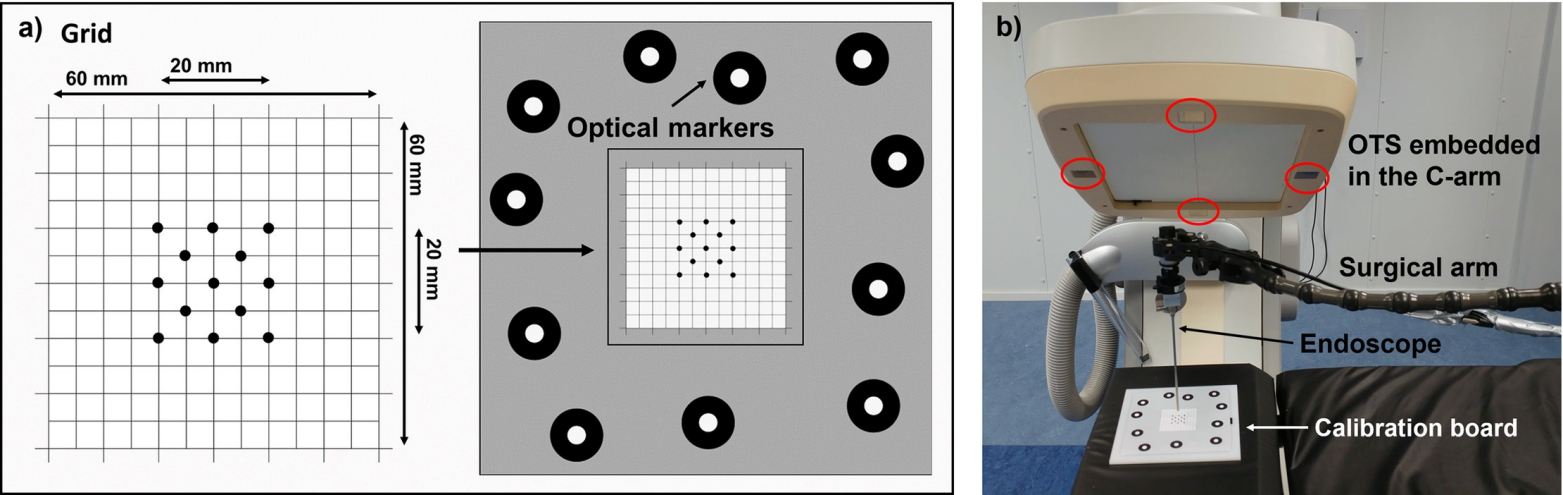
a) Custom-made grid designed for studying the accuracy of the image overlay on the endoscopic view. b) The endoscope was held in a perpendicular position with respect to the grid by means of a surgical arm.

A custom-made grid was designed to test the accuracy of the image overlay on the endoscopic view (Fig 7A). Thirteen stainless steel spheres, with a diameter of 2 mm and a tolerance of 5 μm, were incorporated in the central 20x20 mm of a 60x60 mm grid. A CBCT image of the grid was acquired and the spheres, manually segmented from the CBCT, were overlaid on the endoscopic view. Eleven optical markers were placed on the sides of the grid, allowing tracking of the motion of the grid and adjusting the CBCT position according to grid motion.

The accuracy of the CBCT overlay was tested at distances of 5, 10, 15, 20, 25 and 30 mm from the grid, covering common working distances of the endoscope in neurosurgical skull base procedures. At each distance, the grid was repeatedly photographed with the endoscope, while manually moving the grid to obtain at least 100 positions, covering the entire endoscopic field of view. The grid was kept perpendicular to the straight line of sight of the endoscope, which, in turn, was held in position by a surgical arm (Fig 7B). Endoscopic images were segmented, detecting centres and radii, measured in pixels, of the real spheres and of the overlaid spheres segmented from the CBCT. The error in pixels was converted to millimeters and defined as the target registration error, TRE. For the conversion from pixels to millimeters, the ratio between the diameter of the spheres in the endoscopic image (in pixels) and the real dimension of the spheres (in mm) was used. This conversion is then computed by:

$$TRE\;[mm]=TRE\;[pixel]\times \frac{{⌀}_{Sphere}[mm]}{{⌀}_{Sphere}[pixel]}.$$

### Statistical analysis

The one-way ANOVA with Tukey-Kramer post-hoc analysis was used for statistical analysis of TRE distributions. Results are presented as means with corresponding standard deviations and medians with interquartile ranges.

## Results

**Fig 8:**
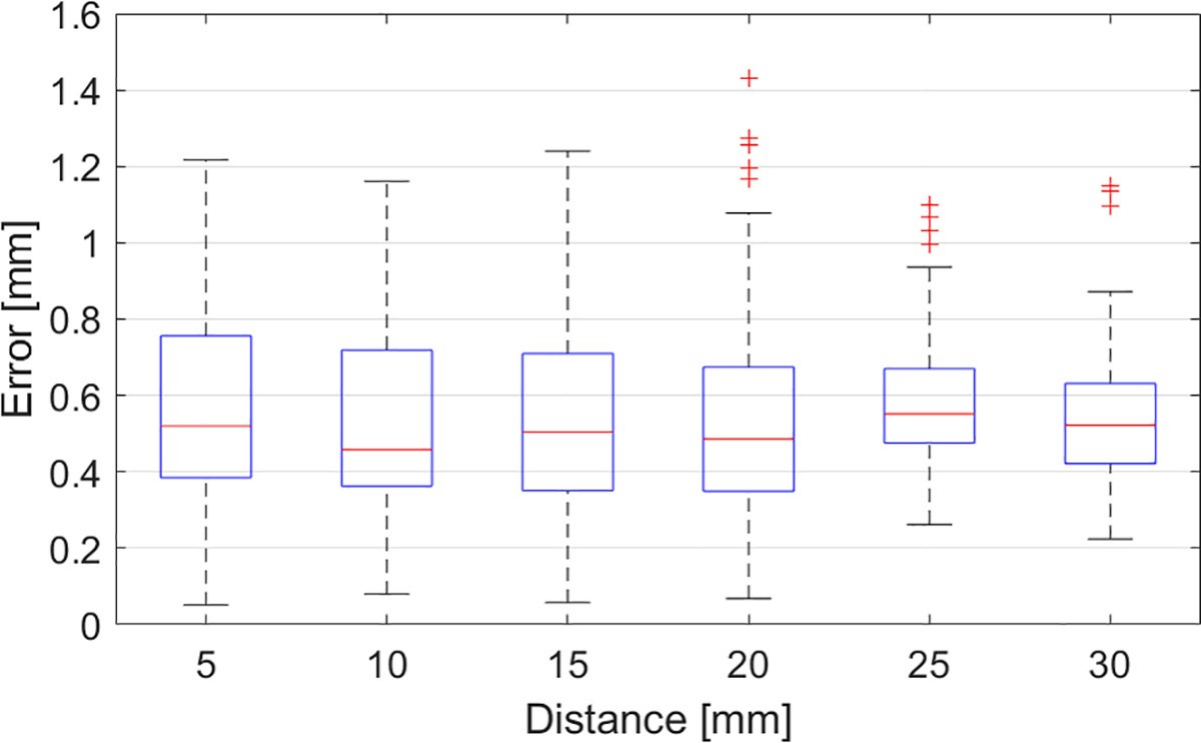
Boxplots of the errors in the image overlay as a function of the distance of the endoscope from the grid.

**Fig 9:**
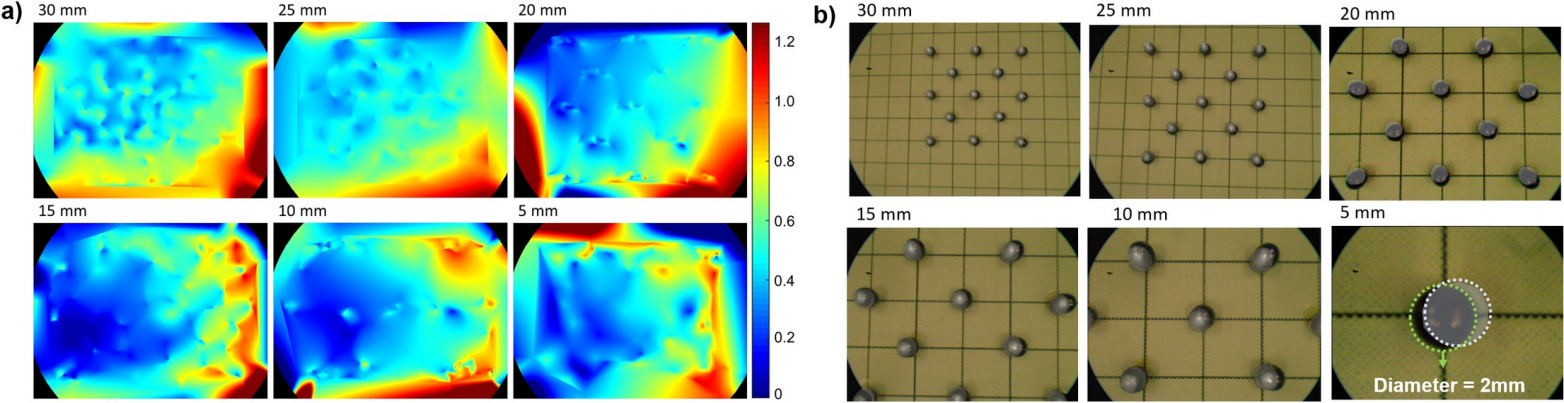
a) Error distribution, expressed in mm, of the image overlay on the endoscopic view at several distances between the endoscope and the grid. Blue represents areas with lower TRE, and red indicates areas with larger TRE. b) Steel spheres segmented from the CBCT and overlaid on the endoscopic view at several distances between the endoscope and the grid.

Overall TRE was 0.55±0.24 mm, with a median of 0.51 mm and interquartile range of 0.39–0.68 mm (Fig 8). Mean and standard deviation, along with median and minimum and maximum values for each distance were calculated using 100 data points. The mean and median values were all notably close to 0.5 mm. The variation (spread) of the error for each distance slightly increased as the endoscope moved closer to the grid, but no significant difference in the mean and median TRE between the distances tested was found (p = 0.37). Furthermore, the measured maximum error was 1.43 mm (outlier). Fig 9A shows the heat maps of the TRE distribution on the endoscopic view at several distances between the endoscope and the grid. The maps show a lower TRE in the central area of the endoscopic image and higher TRE towards the image sides. Also, it should be considered that no image overlay was tested in the corners of the image, since the endoscopic field of view is circular (as shown in Fig 9B).

## Discussion

In this study, we present a novel application for a previously described ARSN system[34]. It has been adapted and developed for endoscopic endonasal skull base surgery with overlay of the intraoperatively acquired CBCT images to create an augmented reality endoscopic view. Sub-millimeter accuracy in CBCT image overlay on the endoscopic view was achieved.

The utility of surgical navigation in endoscopic endonasal skull base surgery is well established [1–6, 10–15]. Most commercially available navigation systems employ a contour-based registration protocol, where a laser pointer is used to identify the skin surface, which is then co-registered with the preoperative CT or MR images[38]. The general consensus is that accuracy, defined as target registration error (TRE), must be less than 2 mm for accurate navigation. [27, 39]. However, this is not consistently achieved with existing navigation systems. [39–41] Moreover, even if mean TRE values are below 2 mm, it is still likely that part of the range will be > 2 mm, resulting in insufficient accuracy in the surgical setting. Therefore, as proposed by Citardi *et al*., “the next immediate goal for a next-generation surgical navigation platform would be to move TRE to 1.0**˗˗**1.5 mm or, ideally, to 0.6 **˗˗**1.0 mm.” [27]. Surgical navigation system prototypes with image fusion on the endoscopic view, have so far not reached TRE values of such low levels [21, 22, 42, 43]. The use of intraoperative CBCT has been suggested as a solution to this problem, as higher registration accuracy on the endoscopic view can be achieved. [42, 44–47]. With respect to other proposed solutions for image fusion on the endoscopic view, our system presents several advantages. Tracking accuracy is always dependent on a combination of the distances between the tracked markers, the distances between the markers and the cameras and the resolution of the cameras. Since the cameras integrated in the flat-panel detector are at close distance to the markers, have a high resolution and a fixed relation to each other, we can track the endoscope with high accuracy [29]. The accuracy in co-registration between OTS and CBCT depends on the distance between them, and since the OTS is rigidly integrated in the C-arm, we can achieve a high accuracy in OTS and CBCT co-registration. Also, while there is no change in their relative position, there is no need for repeating the procedure of co-registration prior to each surgical procedure.

The method presented here, achieves a TRE of 0.55±0.24 mm in CBCT image projection, with a median of 0.51 mm and interquartile range of 0.39**˗˗**0.68 mm, independent of the working distance. The maximum error is 1.43 mm (outlier) and this is well below the currently accepted 2.0 mm [27, 39]. Since there is no standardized method for measuring TRE, the results here should be interpreted cautiously in relation to previous publications. Bong *et al*. achieved an accuracy of about 1 mm in their experiments of image overlay on the endoscopic view [21]. Li *et al*. found a TRE of 1.28±0.45 mm [22]. Mirota *et al*. reported a registration accuracy with a mean TRE of 1.28 mm [42]. Citardi *et al*. estimated a target registration accuracy for surgical navigation of 1.5 mm or better [28]. To the best of our knowledge, the TRE presented in this study is the lowest reported error. However, surgical simulations with printed models and cadavers, tests of inter-user variability and clinical studies are needed to confirm the results of this study, since the accuracy achieved in a laboratory setting may decline as a navigation system is translated into clinical practice.

Using augmented reality for surgical navigation has several potential benefits compared to conventional navigation with display of 2D medical imaging on a separate screen. Overlaying segmented anatomical structures from CT or MRI on the endoscopic video stream enables navigation without the use of dedicated instruments and thereby improves workflow, while visualizing sub-surface anatomy [48]. However, it has been shown that although users of AR navigation were able to identify a target more accurately, they were at the same time at risk of inattentional blindness, e.g. failing to identify unexpected targets like foreign bodies or critical complications [49, 50]. This aspect should be incorporated in the further development of this ARSN system and the design of the associated user interface. It is important that the interface provides only the relevant information to the surgeon. Furthermore, in this experimental setup, the skull phantom was fixed. However, the registration and accuracy of the method does not depend on fixation of the head, since the position of the head is tracked by optical markers with real-time updating of its position. The placement of the optical markers must be carefully investigated to avoid interference with the surgical workflow.

The intraoperative CBCT in the ARSN system is primarily performed for registration purposes, and algorithms for fusion with preoperative MRI images must be developed to enable a-priori pre-planning and segmentation of anatomical structures. Alternatively, there are also several potential advantages with acquisition and post-processing of the intraoperative CBCT images. A contrast-enhanced CBCT could potentially be used for segmentation of the carotid arteries or a contrast-enhancing tumor. There is also the possibility to update the imaging during surgery, e.g. to evaluate tumor resection grade or intraoperative changes of anatomy. Fast and accurate segmentation of CBCT images has been performed successfully intraoperatively in the system’s spine surgery application [48].

### Limitations

In this first study of the ARSN endoscope tracking application, our aim was to set up a system to develop algorithms for tracking of the endoscope with high accuracy. However, the study design has several limitations in evaluating the clinical applicability of the results. The use of a flat grid simplified changes of distance between endoscope and target, and provided measurable targets throughout the endoscopic field of view. However, to prove the clinical value of the system, further testing and simulations on anatomical models as well as cadavers are needed.

## Conclusion

In this study we present a novel application for an augmented reality navigation system in endoscopic surgery, with fusion of intraoperative CBCT to the endoscopic view. A mean TRE of 0.55±0.24 mm was achieved, with a median of 0.51mm and interquartile range of 0.39**˗˗**0.68mm. The system shows great potential for clinical use in endoscopic skull base surgery, and further development is warranted.
