# Correction: Fusion of augmented reality imaging with the endoscopic view for endonasal skull base surgery; a novel application for surgical navigation based on intraoperative cone beam computed tomography and optical tracking

**DOI:** 10.1371/journal.pone.0229454

**Published:** 2020-02-13

**Authors:** Marco Lai, Simon Skyrman, Caifeng Shan, Drazenko Babic, Robert Homan, Erik Edström, Oscar Persson, Gustav Burström, Adrian Elmi-Terander, Benno H. W. Hendriks, Peter H. N. de With

The images for Figs 2, 3, 4 and 5 are incorrectly switched. The image that appears as Fig 2 should be Fig 3, the image that appears as Fig 3 should be Fig 4, the image that appears as Fig 4 should be Fig 5, and the image that appears as Fig 5 should be Fig 2. The figure captions appear in the correct order.

**Fig 2:**
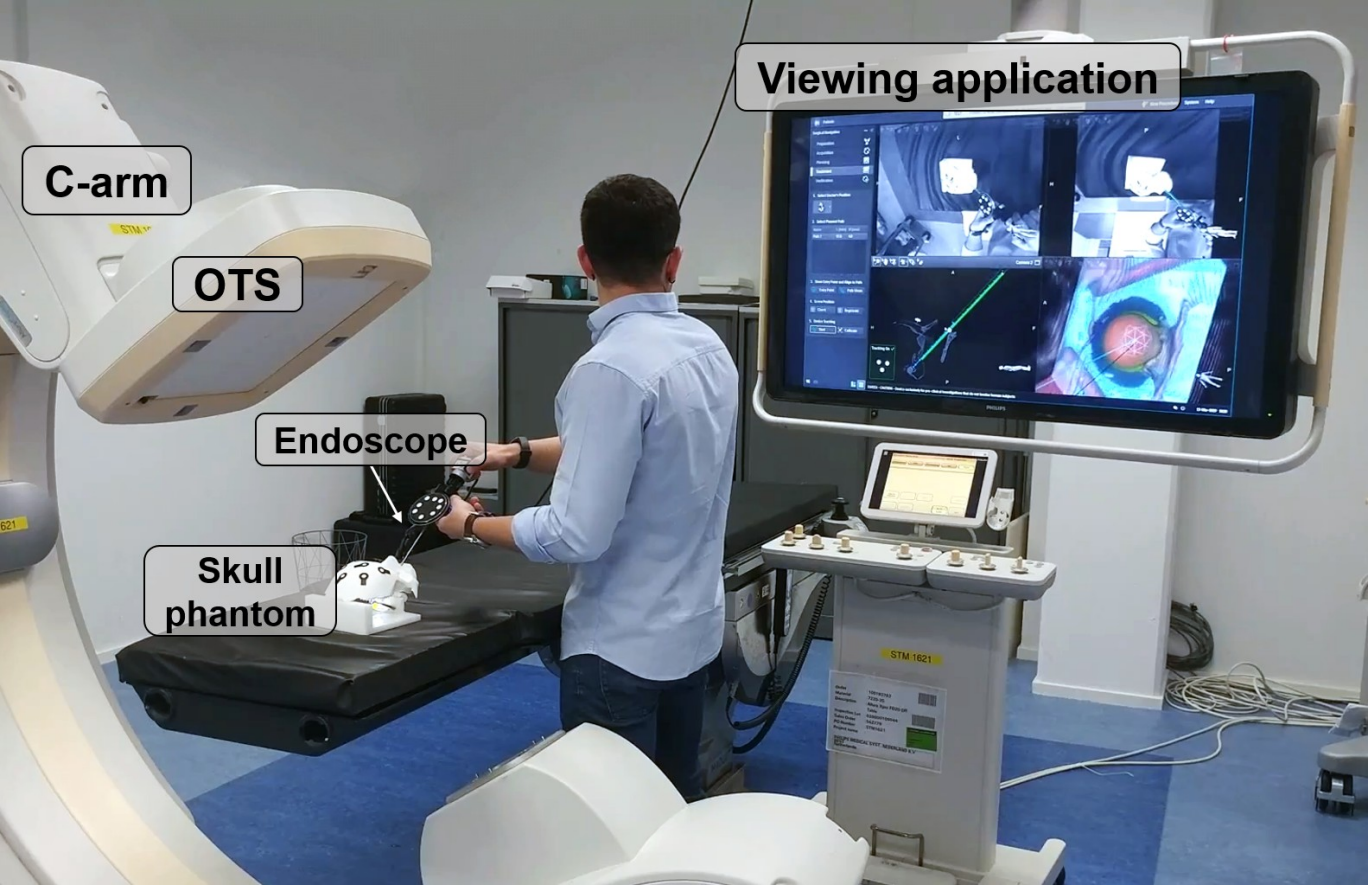
Experimental setup for the study on the skull phantom.

**Fig 3:**
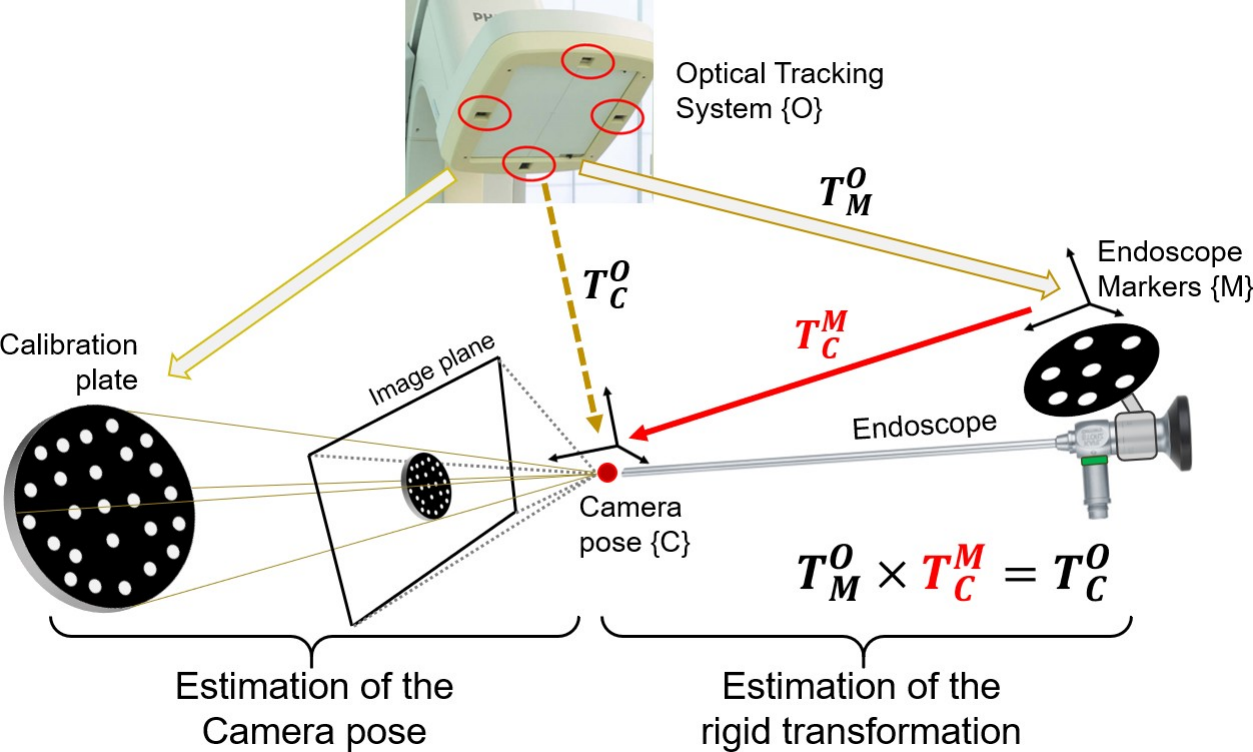
Hand-eye calibration with a moving calibration plate.

**Fig 4:**
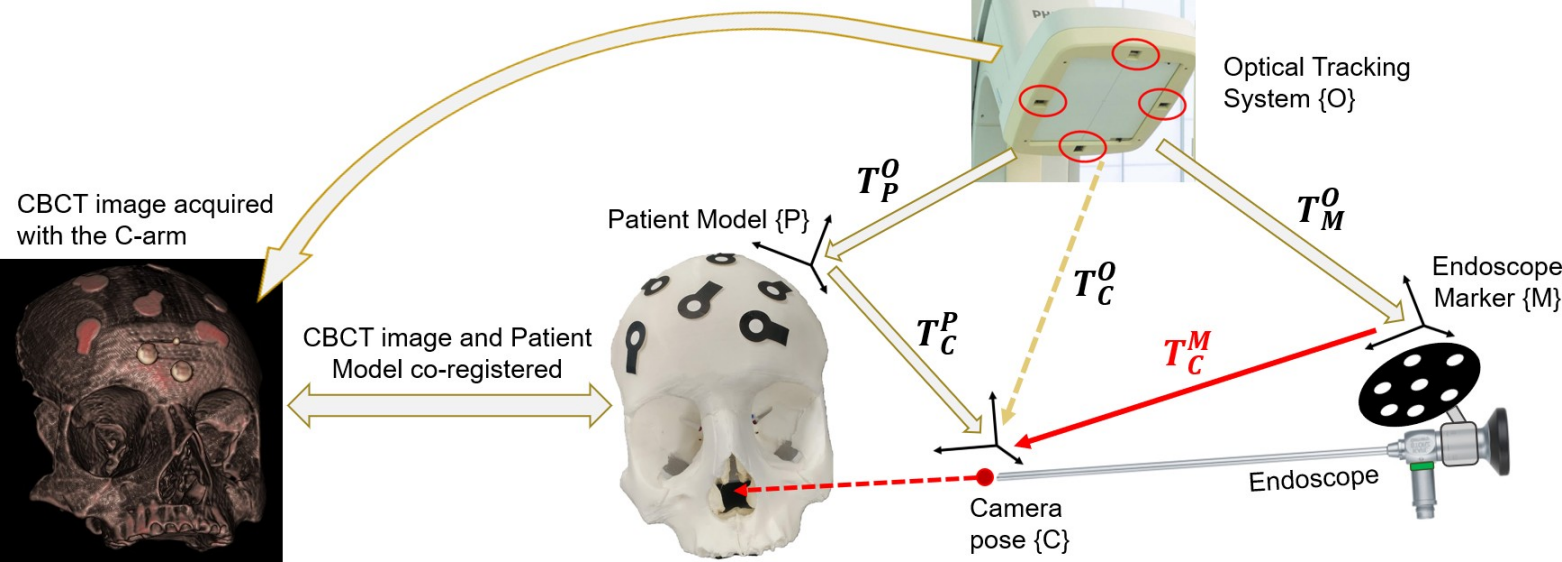
Relationship of the frame transformations.

**Fig 5:**
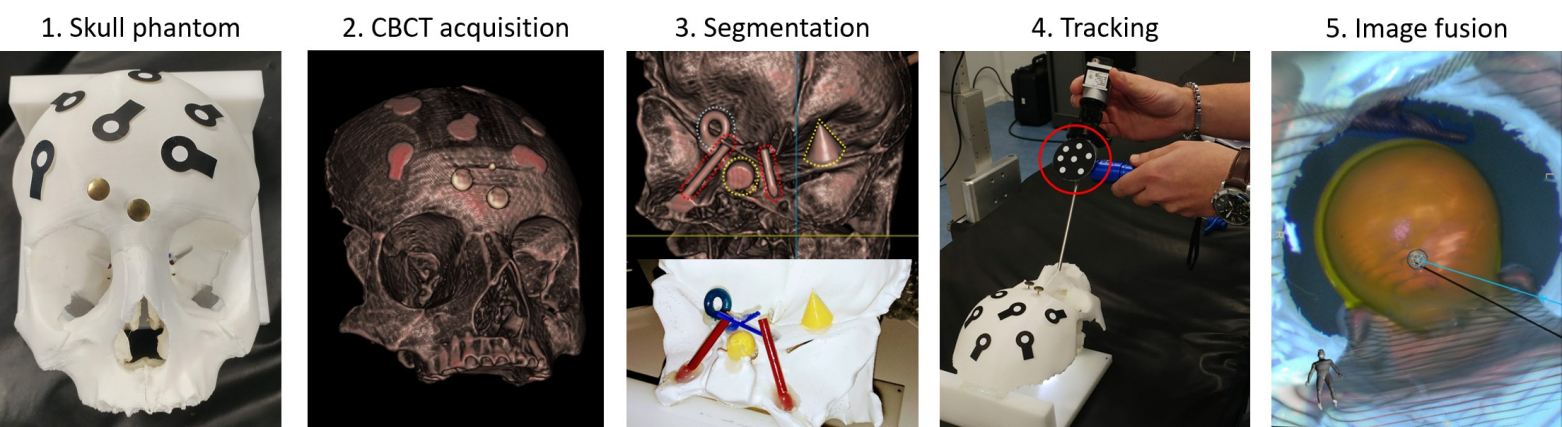
The workflow in a surgical scenario. Overall performances of the image fusion system were evaluated on a plastic skull phantom with a realistic representation of the nasal cavity and adjacent skull base anatomy, including vessels, nerves and the pituitary gland. 1. The skull phantom with optical markers on its surface was positioned on the surgical table. The 3D position of the optical markers was detected by the OTS of the navigation system, to create a VRG for tracking of the phantom’s motion. 2. A CBCT image, co-registered with the 3D position of the optical markers (VRG) was acquired. 3. Anatomical structures of interest were manually segmented from the CBCT image. 4. The endoscope, automatically recognized and tracked by the OTS, was placed in the nasal cavity of the phantom. 5. Segmented structures at the base of the skull were augmented onto the live endoscopic image. The augmented endoscopic view, together with anatomical views to guide the surgeon inside the nasal cavity, were displayed.
